# The Correlation of a Corporate Culture of Health Assessment Score and Health Care Cost Trend

**DOI:** 10.1097/JOM.0000000000001305

**Published:** 2018-02-19

**Authors:** Raymond Fabius, Sharon Glave Frazee, Dixon Thayer, David Kirshenbaum, Jim Reynolds

**Affiliations:** HealthNEXT, Philadelphia, PA (Dr Fabius, Thayer, Kirshenbaum, Dr Reynolds); Frazee Research & Consulting, LLC, Beaufort, NC (Dr Glave Frazee).

**Keywords:** corporate health assessment, culture of health, health scores, health care trend, risk reduction

## Abstract

**Objective:**

Employers that strive to create a corporate environment that fosters a culture of health often face challenges when trying to determine the impact of improvements on health care cost trends. This study aims to test the stability of the correlation between health care cost trend and corporate health assessment scores (CHAS) using a culture of health measurement tool.

**Methods:**

Correlation analysis of annual health care cost trend and CHAS on a small group of employers using a proprietary CHAS tool.

**Results:**

Higher CHAS scores are generally correlated with lower health care cost trend. For employers with several years of CHAS measurements, this correlation remains, although imperfectly.

**Conclusion:**

As culture of health scores improve, health care costs trends moderate. These findings provide further evidence of the inverse relationship between organizational CHAS performance and health care cost trend.

Many occupational health professionals’ roles have evolved or expanded to address the strong connection between workforce health, wellbeing, and safety, and their impact on occupational health.^1^ In addition, these professionals must establish ways to measure progress over time and to justify investments in workforce health in an environment where up to 84% of the full-time workforce has at least one chronic disease or is overweight.^2^ This paper aims to contribute to these efforts, and in particular, to assist corporate physicians and wellness leaders in meeting these demands.

Over the last few decades, corporate health has become much more comprehensive. Traditional occupational health and safety efforts have incorporated workers’ compensation and occupation-related disability management. More recently, occupational health professionals have also been asked to establish efforts to apply primary, secondary, and tertiary preventive services to the workforce and their dependents. Health executives are expected to keep workforces healthy and productive with sustainable and cost-effective programs. Yet, to be successful, companies must build health and safety into the mission, vision, and values of the organization. Adding programs is not enough. The famous quote attributed to Peter Drucker – “culture eats strategy for breakfast” – emphasizes the need to create a work environment where employees and their family members are more likely to make the healthy choice on both a conscious and unconscious basis. Companies have achieved cultures of safety. Now it is time to achieve cultures of health.

How does one define an organizational culture of health? Healthy corporate cultures have a workforce with less illness and fewer unhealthy behaviors. So, employers with “cultures of health” should spend less on health care, without the need to reduce benefit services or shift more costs to their employees. It is feasible to measure a population's culture of health using medical and pharmacy claims information, health appraisals, biometric screenings, and other sources to calculate and track their collective illness burden and risk factors. This may be expressed as reductions in the collective illness burden of employees and their family members, as well as reduced health care cost trend.

Yet, cultural transformation often requires a systematic approach that addresses drivers of culture change, as well as an organizations’ comprehensive efforts to put in place and measure a broad array of coordinated changes to improve health. Measuring corporate cultures of health is a recent and evolving development with significant challenges. As there are a long list of determinants of health, this measurement must be comprehensive, recognizing the influence that work itself has on health as well as health benefit design, workplace environment, and company policies. Measurements must also be meaningful and practical if organizations are going to be willing to apply the resources required.

Employers can measure the health of their culture using one or more of the tools developed to provide a corporate health assessment score (CHAS). Examples of these tools include the Centers for Disease Control and Prevention healthy worksite assessment tool and the on-line self-assessment developed by the Health Enhancement Research Organization in coordination with Mercer (the HERO Scorecard). Two other such tools are the Employer Health Opportunity Assessment™ (EHOA™) and Employer Assessment 50™ (EA50™). The EHOA and EA50 are proprietary culture of health and wellness assessment tools that measure elements that can contribute to a culture of health utilizing data collected via document review, workplace observational site visits, and interviews with senior leadership, management, and employees.

This article tests the stability of the correlation between health care cost trend and scores that measure the culture of health by extending the work by Goetzel et al.^3^ The seminal work by Goetzel et al^3^ demonstrated that another CHAS tool, the HERO Scorecard, was predictive of future health care cost trend. Our hypothesis is that the health care cost trend of companies achieving higher CHAS scores will be lower than companies with lower CHAS scores using data from employer companies that implemented the EHOA/EA50. Moreover, by implementing against a multiyear strategic plan and using simulation, companies can predict the impact of CHAS on future health care cost trend. This has significant implications for financial planning and establishing reserves for covering health care costs.

## CORPORATE HEALTH ASSESSMENT USING THE EHOA/EA50

Corporate health assessments vary in design, but all have the ultimate intention of scoring how an organization is doing in terms of their populations’ health, their corporate health policies, and programs. To illustrate what one corporate health assessment includes, we describe the EHOA/EA50 developed by HealthNEXT™.

Following a similar rigorous research process as done by the author Jim Collins’ from the business book “Good to Great,”^4^ HealthNEXT studied benchmark organizations who had flattened their health cost inflationary trend over many years (in some cases even decreased their total health care spending as a result). The intent of this research was to determine how these critical few employers had achieved success, so that a common framework and methodology could be developed to assist other companies in their pursuit of a culture of health.

In addition, all existing publicly available tools and award standards were studied and incorporated. These included the Centers for Disease Control and Prevention Worksite Assessment Tool, the standards for recognition established by the NBGH Healthy Lifestyles Award, the C. Everett Koop National Health Award, the HERO Employee Health Management Best Practices Scorecard, and the ACOEM Corporate Health Achievement Award. As a result, 218 elements that could contribute to a healthy corporate culture were identified and then organized into 10 categories. The 10 categories, each with an example of included elements were as follows:

- Leadership and management – leaders champion the organizations pursuit of health;
- Marketing and communications – promotion of the value of being healthy;
- Data warehousing – integration of multisource data to allow identification of the illness burden across the health continuum;
- Health and wellness plan design – having a multiyear strategic plan designed to improve the health of the workforce and their families;
- Environment – workplace campus is smoke-free;
- On-site health activities – there is a health clinic or health coaches on-site;
- Health and wellness activities – support for those with chronic conditions designed to mitigate the potential disease complications;
- Incentives and benefit design – provide economic advantages for establishing a medical home;
- Engagement and navigation – tracking the waterfall of programs from invitation, to participation, to completion;
- Vendor integration – evidence of separate vendors working in concert.

In addition, each element identified was assessed against key indicators of awareness, acceptance, and use, supported by rigorous tracking and analysis. This takes corporate health assessments beyond the “what” to the “how.” Leveraging peer-reviewed literature and expert opinion, these elements and categories were weighted to allow for a perfect score of 1000 points. Finally, recognized benchmark culture of health organizations was scored using this assessment process, allowing the establishment of a target benchmark score. On average, each benchmark employer had implemented approximately two-thirds of the 218 potential activities with benchmark scores between 650 and 700 points, out of the possible 1000. While most other efforts to score an employers’ culture of health are paper based and often self-assessments, the EHOA/EA50 assessment process includes the following steps to compare the organization's current state against benchmark “culture of health” employers:

- Health-related claims and benefit design review
- Workplace sites visits of a representative sample of locations
- 360-degree interviewing of leadership, management, and employees

Each assessment has two or three “trained reviewers” with significant health care backgrounds - one of the assessors being a physician executive. The assessment process utilizes a software program that allows each reviewer to enter their evaluation for each of the 218 elements across the 10 categories. The software program then generates a score and identifies any differences in the assessment between reviewers. Discrepancies are then reviewed by the team and a consensus score determined to minimize inter-reviewer variability.

The CHAS tool was then adjusted to accommodate mid-sized employers (200 to 2000 employees); Mid-sized employers often lack items that are scored for larger companies such as on-site health clinics or cafeterias. By reducing the number of potential action items to 50 from 218, but maintaining the integrity of the categories and thresholds, these smaller organizations can have their culture of health assessed and scored against a benchmark of mid-sized organizations recognized for their cultures of health. These best practice companies also scored in the 650 to 700 range, validating the crosswalk from the large employer EHOA. HealthNEXT calls this version the Employer Assessment 50 or EA50.

## PREVIOUS STUDIES EXAMINING CORPORATE HEALTH ASSESSMENT SCORES AND HEALTH CARE COST TRENDS

Previous studies have asserted that incorporating universal health and safety metrics when measuring corporate performance would help employers overcome many of the barriers to establishing a culture of health in the workplace.^5,6^

Although not designed as a corporate health assessment per se, one of the first comprehensive sets of measures used in the United States was the Dow Jones Sustainability Index (DJSI).^7^ Launched in 1999, the DJSI was designed to measure corporate sustainability and create an index of best-in-class organizations to inform the investment community. Key social, economic, and environmental factors that can contribute to an organizational culture of health, safety, and wellbeing are included on the DJSI. Another pioneer in the measuring cultures of health was the American College of Occupational and Environmental Medicine's (ACOEM) Corporate Health Achievement Award (CHAA), which also includes a robust assessment tool. Using a 1000-point scale, this scorecard helps award reviewers determine organizations with exemplary healthy, safety, and environmental programs.^8^

Concepts from the DJSI and CHAA were recently incorporated into the ACOEM-proposed Integrated Health and Safety Index (IHS Index) to recognize to the contributions health and safety programs make to corporate sustainability across economic, environmental, and social dimensions.^9^ Containing 40 key metrics, the IHS Index allows for a wide variety of approaches to integrating health and safety in the workplace. Internationally, other indices such as the Healthy Company Index in South Africa^10^ and Arogya World's Healthy Workplace awards in India^11^ have also being developed.

One of the most widely used workplace health scorecards was developed in 2006 by the Health Enhancement Research Organization (HERO) Think Tank Task Force for Metrics.^12^ The resulting HERO Employee Health Management Best Practices Scorecard in Collaboration with Mercer® (HERO Scorecard) includes a self-assessed inventory of workplace health promotion practices in six domains, including strategic planning, organizational and cultural attitudes, programs delivered, program integration, participation strategies, and measurement/evaluation strategies.^3,12^ The HERO Scorecard is, to date, the only nationally recognized scorecard of employee health with published studies correlating performance on the scorecard with health care cost trend reductions.^3^

Taken together, prior research emphasizes the value of improved culture of health, expressed in part by reduced health care inflation. Research also points to a clear need for measurement and consistency across organizations in order to benchmark performance on dimensions thought to be important in measuring a culture of health.

## PURPOSE

The purpose of this research is to test the stability of the correlation between health care cost trend and scores that measure a corporate culture of health utilizing comprehensive proprietary culture of health assessment tools (EHOA or EA50). We aim to contribute to the increasing evidence of a relationship between CHAS and corresponding health care cost trends. This study is designed to determine whether variations in CHAS are correlated with differences in health care cost trend. To achieve this aim, we identified a group of employers that both (1) had an annual CHAS and (2) provided annual medical and pharmacy cost trend rates. We then analyzed the relationship between CHAS and variations in annual health care costs, comparing organizations who scored higher versus those scoring lower on the culture of health continuum.

## METHODS

During the period of 2011 to 2016, we developed, validated, and implemented the EHOA and EA50, proprietary CHAS tools designed to score an organizations’ culture of health against benchmark culture of health employers. We collected data from the first 21 sets of annual organizational scores where medical and pharmacy cost data were available to determine whether there was a relationship between CHAS and trends in health care expenditures. We then used the data to measure the correlation between the EHOA or EA50 score and annual cost trend across all participating organizations as well as within organizations with several years of CHAS.

### Study Sample

Data for this study were collected from 21 sets of annual organizational CHAS and health care cost trend data. For simplicity, these are referred to as organizations from this point. Four of these organizations had more than 1 year of participation. A total of 12 unique companies providing a total of 21 sets of annual CHAS/health care cost trend data points are included.

Study organizations varied by size and industry. Organizations ranged in size from medium to large (2000 to 350,000 covered lives), and in total, represent over 1 million total covered lives. The mean organization size was 87,458 covered lives with a median size of 20,000 lives.

### Measures

Both CHAS and health care cost trend are continuous variables with a possible range of 0 to 1000 for CHAS and an infinite percentage increase or decrease range for health care costs.

Annual health care cost trend was measured as the total increase year-over-year in total costs for all monies paid to health care providers for the organizations’ covered population by both employers and employees. These costs included both medical (inpatient and outpatient), prescription drug costs, deductibles, copayments, and coinsurance. Health care cost data were obtained from health and pharmacy benefit management carrier reports provided to the organization. Those organizations with multiple carriers had a composite trend calculated by weighting carrier-level trend by the number of lives covered by that carrier. Organizations with significant changes in their health benefit design during the study period were excluded.

### Analysis

The relationship between CHAS and health care cost trend addressed two key questions:

- Is having a higher CHAS correlated with a lower health care cost trend?
- Do relationships between CHAS and health care cost trend remain intact over time for organizations completing continuous years of assessments?

The strength of the relationship between CHAS and health care cost trend was tested using bivariate correlation analysis. Spearman rank-order correlation coefficient, rho (*P*), was used to test the strength of the correlation due to the non-normal distribution of CHAS in the sample. Linear regression was used to test the mean impact of CHAS on health care cost trend.

For the second question, we explored the temporal change in CHAS and health care cost trend for those organizations with multiple years of health assessment score and health care cost trend observations.

All analyses were conducted with IBM SPSS for Windows, Version 24.0 (IBM Corp., Armonk, NY).

## RESULTS

### Study Sample Characteristics

**FIGURE 1:**
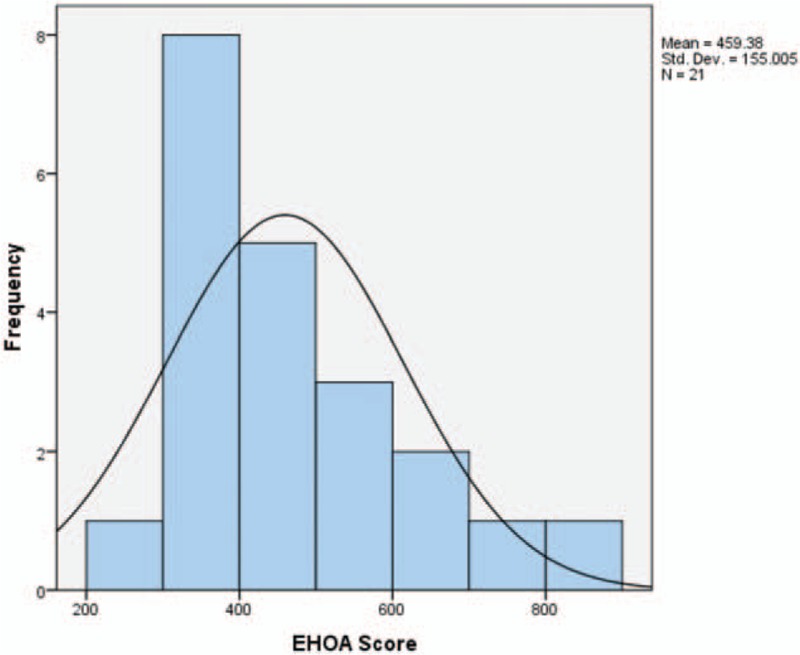
Corporate health assessment score frequency distribution.

**FIGURE 2:**
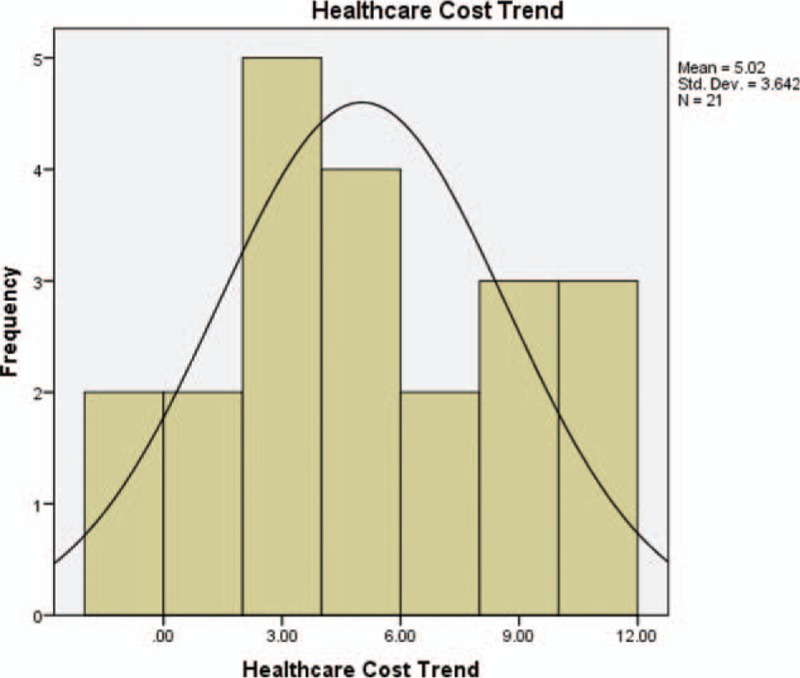
Health care cost trend frequency distribution.

We identified 21 health score assessments completed between 2011 and 2016 for organizations with health care cost claims data available for the same calendar year. Table 1 summarizes the year of CHAS completion as well as the number of covered lives for each year, and the mean, minimum, and maximum for CHAS and health care cost trend each year. One-third of organizations completed the scorecard in 2015 (33.3%). For the entire sample, the average score was 459 and the average health care cost trend was 5.0%, although this varied by year and organization. Figures 1 and 2 show the distribution of CHAS and health care cost trend for the full sample. CHAS scores are skewed toward the lower end of the continuum, while health care cost trend is more normally distributed. Because two types of CHAS tools are included (EHOA and EA50), we did extensive testing to determine if there was any impact from inclusion of both tools for all analyses conducted. No relationships were found. The correlation testing the effect of which the tool used was very weak (nonstatistically significant correlation of −0.299).

### Relationship between CHAS and Health Care Expenditures

**FIGURE 3:**
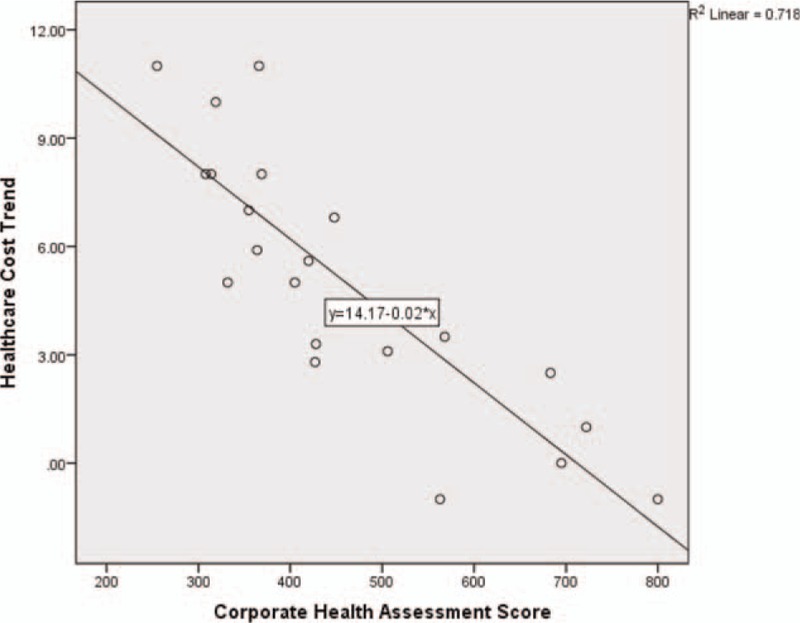
Corporate health assessment score by health care cost trend.

In the analysis of relationship between CHAS and health care expenditures, bivariate correlation results show a strong correlation (*r* = −0.848, *P* < 0.001) for organizations with higher risk scores to have lower annual health care cost trend, as the value is negative and close to −1. The data points are clustered closely along a negative sloping line as shown in Fig. 3.

As several organizations had multiyear trend-EHOA score observations, we also tested for auto-correlation. The Durbin Watson statistic of 2.073 is within the range considered normal.

From the 21 data points generated, we attempted to predict health care cost trend from CHAS using simple linear regression analysis and least squares regression weighted by the number of covered lives. The results of the simple linear regression equation, Health care cost trend = 14.171 – (−0.020) (CHAS), found a statistically significant relationship between CHAS and health care cost trend with an *r*^2^ of 0.718. A least squares regression model weighted by the number of covered lives reduced the *r*^2^*r* to 0.704.

Using the more conservative weighted least squares regression model, we are 95% confident that the slope of the true regression line is somewhere between −0.020 and −0.010, and thus assume that for each point increase in CHAS, the average percent health care cost trend decreases somewhere between −0.020 and −0.010. We also tested the impact of the type of CHAS tool used in the model. This addition was nonsignificant so was not included in the final model for parsimony.

**FIGURE 4:**
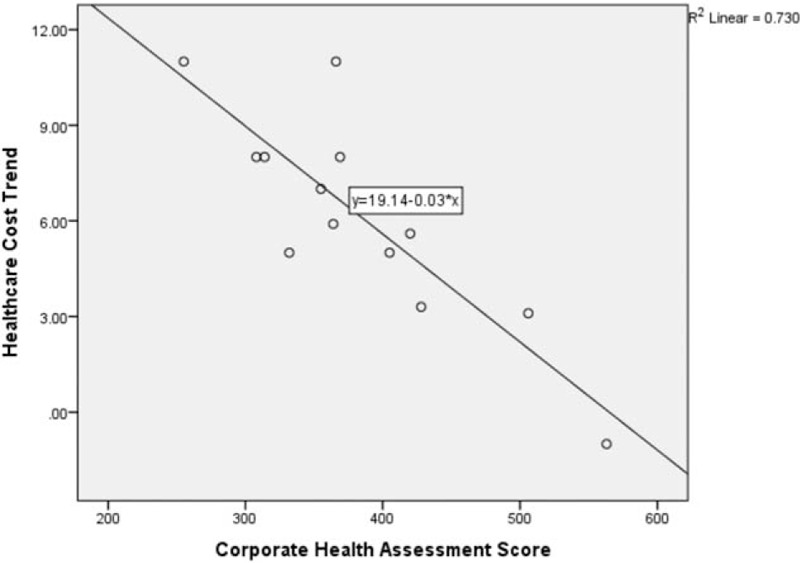
Corporate health assessment score and health care cost trend organizations with multiple years of data.

When examining only those organizations with multiple years of corporate health assessments and health care cost trend, we find a similar, albeit slightly stronger *r*^2^ (.730), as illustrated in Fig. 4.

**FIGURE 5:**
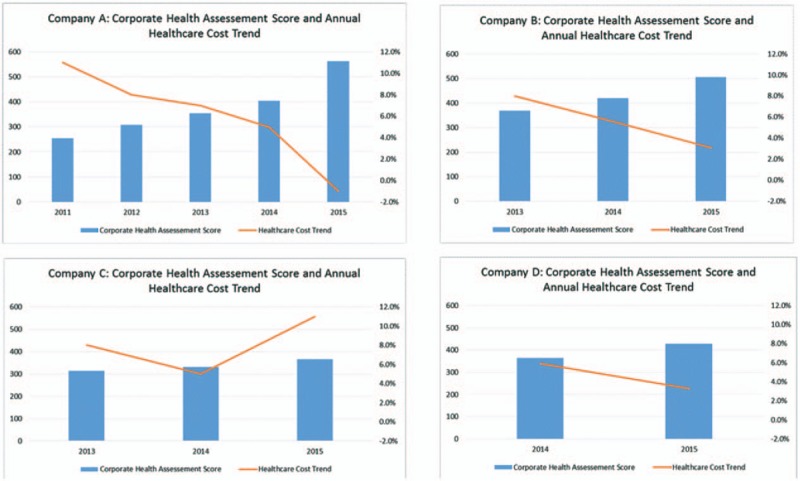
Corporate health assessment score and health care trend by organization.

CHAS and health care cost trend for each organization are also shown in Fig. 5. It is evident that although the assessment score is highly correlated with health care cost trend, this relationship is imperfect. For Company C, scores show a decline in health care cost trend from 2013 to 2014 but an increase in 2015, even while their CHAS continued to improve incrementally over the 3-year period from 314 to 332 to 366.

From the 13 points generated for this subset of organizations with multiple years of data, we conducted simple linear regression analysis. We found a statistically significant relationship between CHAS and health care cost trend with an *r*^2^ of 0.730 and a confidence interval indicated that the true regression line is between −0.048 and −0.020. Thus, we can infer that a 21 to 50-point increase in CHAS score would predict a 1% decrease in health care cost trend. Regression models were also run controlling for the effect of time, but this addition was not statistically significant.

## DISCUSSION

**FIGURE 6:**
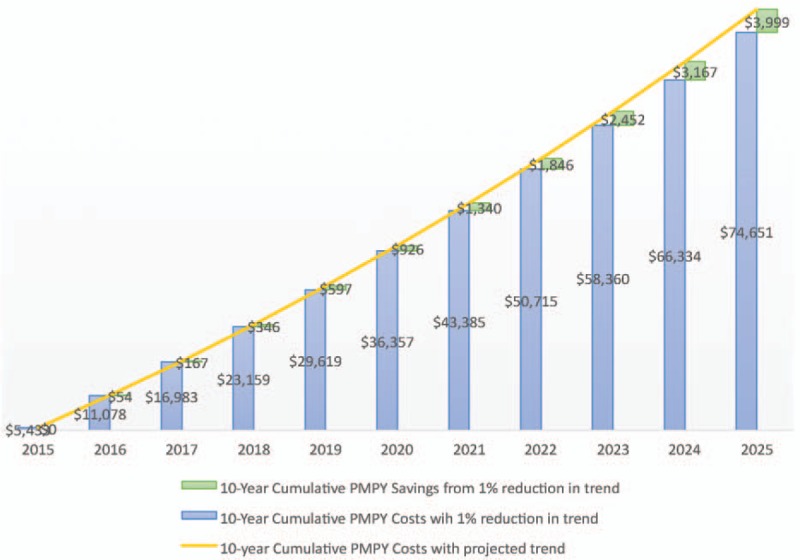
Projected 10-year per member per year savings from 1% annual reduction in health care cost trend.

The correlation of CHAS scores with total health care cost trend is not perfect. This is unsurprising given the number of factors influencing health care costs. A culture of health is an important component, but not the only element at work. Examples of other factors include benefit design, changes to member cost-sharing, provider network and reimbursement changes, significant health care innovation, among others. In general, increasing CHAS scores are linked to lower health care cost inflation. This can add up to a significant amount of money saved. In 2015, U.S. health care spending for private health insurance, which is primarily employer-sponsored in the U.S., saw an average increase of 7.2%.^13^ Per capita U.S. private insurance expenditures in 2015 averaged $5433 per person,^14^ representing a significant cost for employers particularly as only a portion of these persons are employees; the rest being dependents. Forecasted spending growth for private insurance was projected at 4.9% for 2016, averaging 5.6% annually from 2017 to 2019, and then projected at 5.3% annually from 2020 to 2025.^14^ Assuming these projections are reasonably accurate, an average employer would expect per covered member expenditures of $9149 in 2025, a 68% increase in per member expenditures. However, by improving the culture of health and decreasing annual health care trend by even 1%, per member costs would decrease by $3999 over a 10-year period compared with the per member costs for organizations with no change in the trajectory of health care spending (Table 2). Cumulative 10-year per member per year costs using projected trend compared with trend reduced by 1% are also illustrated in Fig. 6.

Implementing best practices in action areas such as comprehensive efforts to reduce health risks such as smoking, high blood pressure, high cholesterol, obesity, as well as assisting all covered lives to receive evidence-based, quality health care can help organizations achieve the 21 to 50 CHAS points needed to attain a 1% decrease in health care cost trend.

It should be noted that these costs reflect national averages and can differ widely by organization, impacted by differences in age, gender, number of dependents, industry type, healthcare benefit design, and regional differences in health spending and utilization, among others.

**FIGURE 7:**
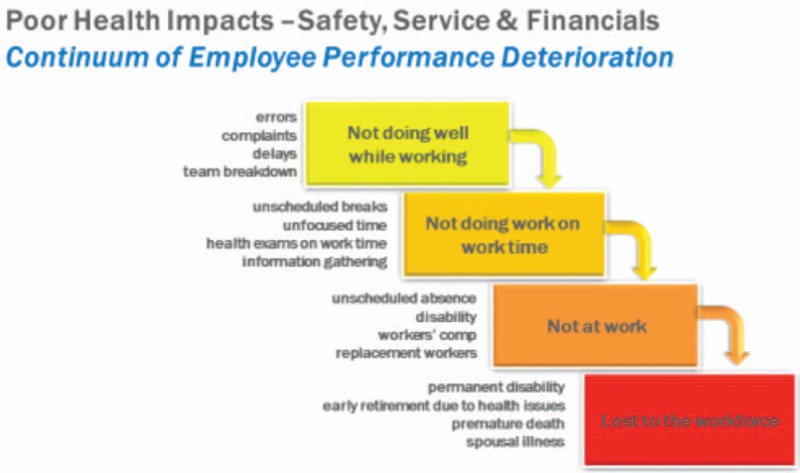
Continuum of employee performance deterioration.

The connection between a healthy workforce and superior marketplace performance is becoming increasingly clear. It is estimated that for every dollar saved in direct health care costs, employers save an extra $2.30 in improved performance or productivity.^15^ Warren Buffet recently sited health care as the “real corporate tax” because of its rate of escalation over the last many years.^16^ Even assuming that annual health care costs increase around 5%, this equates to nearly three times the 1.7% unadjusted 12-month inflation rate,^17^ and more than twice the 12-month inflation rate for private industry wages and salaries.^17^ Thus, direct health care costs are a significant drain on profits even before considering the indirect losses due to reduced productivity. As shown in Fig. 7, when an employee is sick, first they do not perform well at work (presenteeism), then the work is not being completed in a timely matter (delayed production), then the worker becomes absent (absence), and ultimately perhaps even lost from the workforce (disability). All these steps have real impacts on the performance of an organization.

Several studies have shown the benefits of workplace health and wellness programs. These benefits range from financial savings on health care costs, reduced absenteeism, improved health and reduction of illness, reduced employee turnover, and lower rates of work- and nonwork-related disability.

Health care cost savings from health and wellness programs have been shown to range widely. The average found from one meta-analysis of 22 studies was $358 per employee per year, with a 3.27 to 1 return-on-investment.^18^ Other studies have found savings that are even higher.^19–21^ Reduced absenteeism is another area where cost savings are seen, with an average savings in 2009 dollars of $294 per employee per year and return-on-investment of 2.73 to 1.18.

Even more compelling, other studies have shown superior stock performance by organizations who achieve a culture of health as measured by receipt of various health and safety awards such as the American College of Occupational and Environmental Medicine's Corporate Health Achievement Award (CHAA),^22^ the C. Everett Koop award,^23^ and being recognized as high scoring Health Enhancement Resource Organization (HERO) organization.^24^

Some of the best organizational success stories occur when companies integrate the culture of health into how they do business. For example, Lincoln Industries, based in Lincoln, Nebraska, considers wellness to be “hard-wired” into their business. Moreover, they have near universal participation and wellness goals are part of the companies’ annual performance review, at the individual and organizational level.^19^

In practice, organizations who have used CHAS have noted a reduction in high cost cases over time as their employees and covered lives reduce their burden of illness and risk factors. Some examples of areas where improvements have been demonstrated in health and wellness translate into reduced health care cost trend include population illness burden (eg, percentage with chronic illness, percentage with comorbidities, percentage with high cost claims, percentage with disability claims), and risk factor reduction (eg, percentage with 0 to 2, 2 to 4, and 5 or more risk factors). This CHAS attempts to be more comprehensive by incorporating these as well as economic metrics and cultural metrics such as leadership support, analytics, vendor integration as well as employee engagement and satisfaction.

Perhaps most exciting is the potential to reduce costs. Companies that commit to raising their CHAS by 100 points may be able to decrease future health care costs by 1% to 2%. For many companies, this creates a substantial windfall for corporate budgets and improves budget forecasting. More accurate prediction of health care costs could limit the need for reserves and allow for greater investment into infrastructure, research, wages, or investor returns.

## LIMITATIONS

This study is limited by the small number of organizations who have completed a CHAS and had complete health care trend data available. A critical limitation to keep in mind is that the sample size, especially of unique organizations, is small and are a self-selected employer base. The latter is an important distinction, as most of these organizations have had health and wellness programs in place for a least a few years, and have an orientation toward health and wellness as a business advantage that may not be typical of most U.S. companies. In fact, a RAND study sponsored by the Departments of Labor and Health and Human Services found that only about half of the employers who reported having workplace wellness programs formally evaluated program impact.^25^ We suspect that measurement of a culture of health is less common than for individual health and wellness programs. The authors also believe that the “average” employer would score lower and have much higher health care cost trends.

The statistical power constraints of the small self-selected sample size limit the capacity to generalize the conclusions drawn. The authors plan to follow this study with additional published research, as the number of companies participating grows over time.

It is also important to remember that correlation does not imply causation. Health care cost trend can be impacted by many variables beyond the control of an employer's culture of health efforts such as changes in access to care, community medical practice, and benefit design.

This paper should encourage others to pursue the measurement of “cultures of health” and to correlate the various existing efforts such as those developed by HERO, NBGH, the KOOP Award, and ACOEM's own CHAA. Moreover, these measures need to demonstrate tangible value to employers to justify their expense. Ultimately, research should be able to demonstrate that comprehensive efforts to create a “culture of health” produce less illness burden, lower health care and pharmacy costs, less disability, and greater workforce performance.

## CONCLUSION

Organizations that invest in developing a culture of health and wellness demonstrate the ability to bend the health care cost curve. Workplaces with a culture of health and wellness surround employees with an environment, policies, and cues that support making healthy choices on both a conscious and unconscious basis.^26^ At their best, these cultures make healthy choices the easier choice.

A culture of health and wellness leverages multiple population health strategies and focuses not only on employees with chronic illnesses or serious risk factors but also on the currently well. The culture respects the influence that social determinants have on health and encompasses physical, mental, social, intellectual, career, financial, and spiritual wellbeing.^27,28^ Remarkably, employers can provide support for many if not all of these components of wellness.

A great challenge for the corporate health and wellbeing movement has been to provide “proof” that a healthy workforce provides a competitive advantage to employers.^29^ This paper attempts to add another step toward developing an evidence base that workforce health and performance is not just a correlation but may be causally related. Additional research is needed with a larger sample and using other CHAS instruments. The authors hope that others will contribute to this important body of evidence.

In addition, a culture of health measures and tracks changes over time. The measures may vary by organization or program, but the data collected are key to not only demonstrating outcomes but also for making systematic improvements as process and outcomes measures are assessed and analyzed.

What is clear is that reduced health care spending provides dividends far beyond direct health care costs. Research suggests that the total economic impact of instituting an organizational culture of health can be much greater than just bending health-related costs. Among the impacts can be increased productivity and performance, reduced waste, absence and disability, increased employee engagement and loyalty, as well as elevating a company to “employer of choice” status.

The relationship between health and work is bidirectional.^28^ Work affects health, and health affects work. Healthy workers are more productive, have fewer disability days, are absent less, and use fewer health care resources. Given that employees spend the majority of their waking hours in the workplace, this makes sense and also makes the workplace an ideal place to offer programs that support the creation of healthier employees.

It is the hope of the authors that this research contributes to the growing evidence that a healthy workforce provides a competitive advantage in the marketplace. And by doing so it makes it easier for occupational and corporate physician executives to obtain the resources and leadership support necessary to build a sustainable culture of health within their organization.

## Figures and Tables

**TABLE 1:** Sample Organization Characteristics Overall and by Year of CHAS Score Completion

			Corporate Health Assessment Score			Health Care Cost Trend		
Year	Organizations *n* (%)	Total *n* Covered Lives	Mean	Minimum	Maximum	Mean	Minimum	Maximum
2011	3 (14.3)	445,000	592	255	800	3.6%	−1.2%	11.0%
2012	3 (14.3)	82,500	437	308	683	6.8%	2.5%	10.0%
2013	3 (14.3)	190,000	346	314	369	7.7%	7.0%	5.9%
2014	4 (19.0)	205,000	380	332	420	5.4%	5.0%	0.0%
2015	7 (33.3)	560,000	472	366	568	4.2%	−1.0%	11.0%
2016	1 (4.8)	2,000	695	695	695	0.0%	0.0%	0.0%
Total	21 (100.0)	1,484,500	459			5.0%		

**TABLE 2:** Projected per Member Cost Trend and Savings from Decreasing Annual Trend by 1%

Year	Projected Annual Cost Trend Increase	2015 Actual and 2016–2025 Projected) Cost per Member	Projected Cost per Member With 1% Decrease in Annual Trend
2015	7.20%	$5,433	$5,433
2016	4.90%	$5,699	$5,645
2017	5.60%	$6,018	$5,905
2018	5.60%	$6,355	$6,176
2019	5.60%	$6,711	$6,460
2020	5.30%	$7,067	$6,738
2021	5.30%	$7,442	$7,028
2022	5.30%	$7,836	$7,330
2023	5.30%	$8,251	$7,645
2024	5.30%	$8,689	$7,974
2025	5.30%	$9,149	$8,317
10-Year per member total		$78,650	$74,651
10-Year per member Savings from 1% decrease in annual cost trend			**$3,999**
